# Supplementary material for: Barrier Protection and Recovery Effects of Gut Commensal Bacteria on Differentiated Intestinal Epithelial Cells In Vitro
Source: Nutrients. 2020 Jul 28;12(8):2251. doi: 10.3390/nu12082251 (PMC7468801; doi:10.3390/nu12082251)
Supplement: Supplementary file 1 [file nutrients-12-02251-s001.pdf]

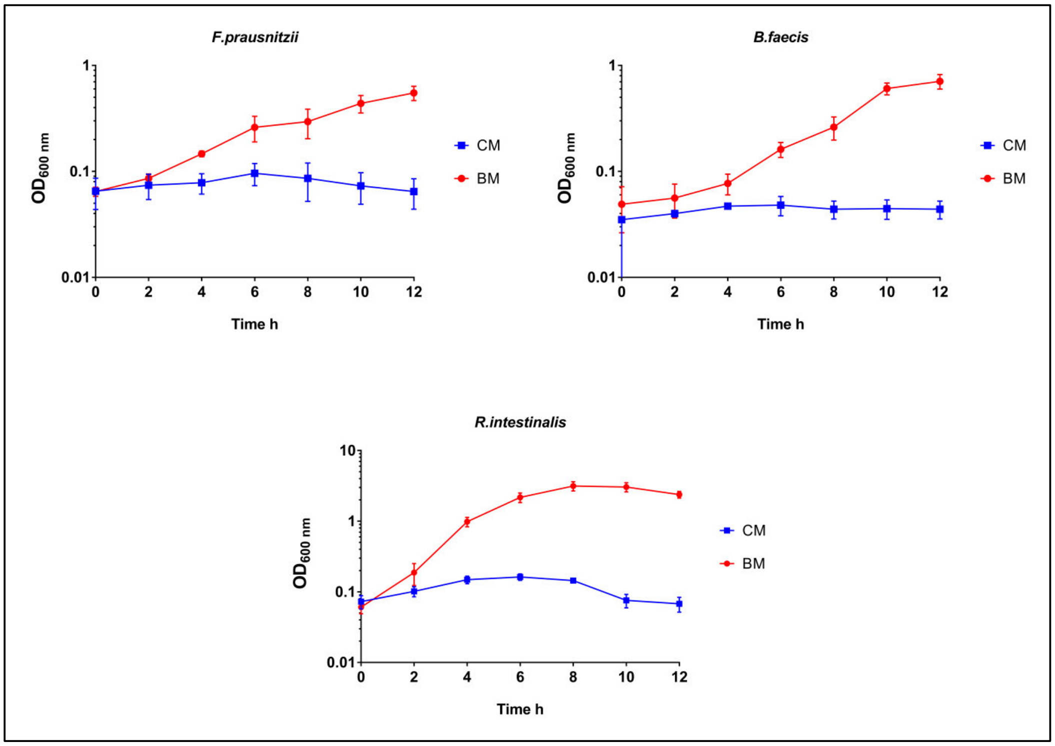


**Figure 1. Viability of the three *commensal bacteria species* in bacteria culture medium and cell culture medium.** *F. prausnitzii, B. faecis* and *R. intestinalis* in stationary phase suspended in anaerobic DMEM (CM) and supplemented YBHI (BM) and incubated anaerobically at 37°C for 12 hours. The optical density (OD600nm) was measured every 2 hours over the period of 12 hours. Results are represented as the mean of triplicate experiments ±SD.
